# Induced Pluripotent Stem Cells derived Muscle Progenitors Effectively Mitigate Muscular Dystrophy through Restoring the Dystrophin Distribution

**DOI:** 10.4172/2157-7633.1000361

**Published:** 2016-09-26

**Authors:** Wen-Feng Cai, Wei Huang, Lei Wang, Jia-Peng Wang, Lu Zhang, Muhammad Ashraf, Shizheng Wu, Yigang Wang

**Affiliations:** 1 Department of Pathology and Lab Medicine, College of Medicine, University of Cincinnati, Cincinnati, OH 45267-0529, USA; 2 Key Laboratory of Functional Proteomics of Guangdong Province, Department of Pathophysiology, Southern Medical University, Guangzhou 510515, China; 3 Department of Pharmacology, University of Illinois at Chicago, Chicago, IL 60612, USA; 4 Qinghai Provincial People’s Hospital, 2 Gonghe Rd, Xining, Qinghai, 810007, China

**Keywords:** iPSCs, Muscular progenitors, Duchenne muscular dystrophy, Dystrophin

## Abstract

**Background:**

Duchenne Muscular Dystrophy (DMD) is a recessive form of muscular disorder, resulting from the dystrophin gene mutations in X-chromosome. Application of embryonic stem cells or adult stem cells has demonstrated the therapeutic effects on DMD through both cell-based and non-cell based mechanisms. In this study, we proposed that Myogenic Progenitor Cells from Induced Pluripotent Stem Cells (iPSC-MPCs) would be more effective in repairing muscle damage caused by muscular dystrophy.

**Methods and results:**

Mouse iPSCs were cultured in myogenic differentiation culture medium and the MPCs were characterized using Reverse Transcription Polymerase Chain Reaction (RT-PCR) and flow cytometry. iPSCs were successfully converted into MPCs, as evidenced by the distinct expression of myogenic genes and cell surface markers. The muscle injury was induced in tibialis muscle of mdx mouse by cardiotoxin injection, and the iPSC-MPCs were then engrafted into the damage site. Firefly luciferase expression vector was transduced into iPSC-MPCs and the *in vivo* bioluminescence imaging analysis revealed that these progenitor cells survived even at 30-days post transplantation. Importantly, histological analysis revealed that the central nuclei percentage, as well as fibrosis, was significantly reduced in the iPSC-MPCs treated muscle. In addition,the transplantation of progenitor cells restored the distributions of dystrophin and nicotinic acetylcholine receptors together with up-regulation of pair box protein 7(Pax7), a myogenic transcription factor.

**Conclusion:**

iPSCs-derived MPCs exert strong therapeutic effects on muscular dystrophy by restoring dystrophin expression and acetylcholine receptor distribution.

## Introduction

Duchenne Muscular Dystrophy (DMD) is a rapidly recessive form of muscular dystrophy. Although the incidence of this disorder is 0.027% only in young males, the survival rate and the quality of life in patients is very poor. Particularly, proximal muscle weakness in the legs and pelvis, the early symptom of DMD, will spread to the arms, neck and other areas, and eventually result in respiratory paralysis to threaten life.

During pathological development of DMD, the major cellular architecture change is disruption of Dystrophin-Glycoprotein Complex (DGC), which spans the muscle plasma membrane and provides a mechanical linkage between laminin in the extracellular matrix and actin in the intracellular cytoskeleton [1]. Indeed, focal lesions in the plasma membrane appeared as a pathological change in DMD muscular fibers [2], and this membrane abnormality not only compromises the contractile function but also jeopardizes the viability of skeletal muscle through disturbing the activity of signaling complexes and channels. Actually, it has been reported recently that Ca^2+^ disorder is a final common pathway for mediating cellular necrosis [3], resulting in progressive muscle wasting and premature death [4]. Dystrophin is an important cohesive protein in assembling the dystrophin-glycoprotein complex. In the presence of dystrophin deficiency or dysfunctional mutation, this complex fails to assemble on the sarcolemma, leading to the irreversible loss of skeletal muscle, which is gradually replaced by fibrotic and adipose tissue [5]. Some preclinical trials have been attempted to mitigate DMD through gene or cell-mediated therapies to restore dystrophin expression [6]. In clinical practice, although the onset of symptoms can be controlled to maximize the patient’s quality of life, the current clinical therapeutics cannot reconstitute the regeneration function of skeletal muscle, since the endogenous satellite cell pool is exhausted [7]. Embryonic Stem (ES) cells have demonstrated a great potential for cell therapy against gene-mutated diseases via both cell-based and non-cell based therapeutic mechanisms. Indeed, it has been reported that the working muscle fibers can be generated from ES cells to replace the damaged skeletal muscle cells [8], and these stem cell release protective cytokines and chemokines to reduce inflammation, slowing down the progression of muscular dystrophy [9]. Unfortunately, the ES therapy faces several difficulties including tumor genesis, immuno-rejection, and ethical issues which limit its application in the clinic [10].

Induced Pluripotent Stem Cells (iPSCs) are important source of progenitor cells [11] which can be prepared for transplantation. In this study, we used iPSCs to generate Myogenic Progenitor Cells (MPCS), characterized their phenotype, and investigated their survival and then engraftment into dystrophic muscle tissue. Additionally, pathological and morphological changes were documented on the therapeutic effects of these progenitor cells against cardiotoxin-induced Duchenne muscular dystrophy.

## Materials and Methods

### Cell culture

Mouse adult fibroblasts were isolated from wild type mouse (C57/BL6), and the cell reprogramming was performed by lentiviral transduction with Yamanaka factors (Oct-4, Sox2, Klf4, c-Myc). The reprogramed-iPSCs were then cultured in DMEM, plus 20% FBS and 1000 U/ml LIF (leukemia inhibitory factor; Chemicon, Billerica, MA), 2 mM glutamine (Gibco, Grand Island, NY), 1 mM sodium pyruvate (Gibco), 0.1 mM β-mercaptoethanol (Gibco), and 2 mM non-essential amino acids (NEAA, Gibco). Medium was changed every day until confluent. 0.05% trypsin-EDTA (Gibco) was used to passage cells.

### Preparation of muscular dystrophy mouse model

Mdx mice were purchased from Jackson lab. The colony was maintained by crossing mdx males with mdx females. The present study was in accordance with the Guide for the Care and Use of Laboratory Animals published by the US National Institutes of Health (NIH Publication No.85-23, revised 1996) and the National Research Council Guide for the Care and Use of Laboratory Animals: 8th Edition published by The National Academies Press, 2011, Washington, DC. All animal experiments were approved by the Institutional Animal Care and Use Committee (IACUC) of the University of Cincinnati (Protocol No.06-03-03-01). After anesthesia with ketamine (5 mg/ ml) and xylazine (1 mg/ml), cardiotoxin (10 μM) was intramuscularly administered into the tibialis anterior (TA) and gastrocnemius (GC) muscle to simulate muscular dystrophy. Then the left limb was engrafted with iPSC-MPCs and the right side was injected with PBS, which served as control. The experimental mice were sacrificed at 4 weeks post cell transplantation, and the muscle tissue was harvested for further analysis.

### Preparation of iPSC-derived MPCs

Preparation of iPSC-derived MPCs was performed as previous protocol with modification [12]. Briefly, differentiated iPSCs were dispersed using non-enzymatic dissociation buffer (Sigma-Aldrich, Cat No. C5914), which were then incubated with CD45 antibody and performed manual magnetic-activated cell sorting (MACS, Miltenyi Biotec Inc, San Diego, CA). The CD45- cells were then selected and harvested to perform further MACS, to get CD45--Sca1--Mac- cell population. These cells were then co-stained with FITC-conjugated CD29 antibody (eBioscience, Cat No. 12-9991-81) and PE-conjugated CXCR4 antibody (eBioscience, Cat No. 11-0291-80). FITC-PE double positive cells were then selected and enriched using fluorescence-activated cell sorting (BD FACSAria II).

### Analysis of myogenic gene expression by RT-PCR

Total RNA was extracted from iPSCs-derived MPCs using mRNA extraction kit (Qiagen, Cat. 217004; Valencia, CA) and cDNA was prepared with the miScript^®^ PCR starter kit (Qiagen, Cat. 218193) as described previously [13]. The primers for mouse myogenic genes are listed in Supplemental Table 1. The PCR product was run on a 1% agarose gel to identify the presence of myogenic genes and the GAPDH was treated as internal control.

### Bioluminescence imaging (BLI)

As previously described, the BLI was performed both *in vitro* and *in vivo* [14]. After transduction with vector which contained ubiquitin promoter driving firefly luciferase, BLI of firefly luciferase (Fluc) positive cells on culture plates was used to show whether the Fluc reporter gene was functional. Serially diluted Fluc-overexpressing iPSC-MPCs were seeded on a 24-well tissue culture plate and non-transduced iPSC were used as the control group. Cells in each well were incubated with 0.5 ml 150 μg/ml D-Luciferin (sodium salt; Gold Biotechnology, St. Louis, MO) in PBS and BLI imaging was immediately performed. Bioluminescence was quantified in units of maximum photons per centimeter squared per steridin (p.s^-1^. cm^-2^.sr^-1^). *In vivo*: BLI was performed in Fluc-overexpressing iPSC-MPCs-engrafted mice at day 30 post transplantation under general anaesthesia (isoflurane) inhalation, (4% for induction and 2.5% for maintenance; n=4). Following intra-peritoneal injection of D-Luciferin (150 mg/kg body weight), animals were then placed in the IVIS 200 Xenogen *in vivo* imaging system chamber (Xenogen, Alameda, CA) for imaging using bioluminescence charge-coupled device (CCD) camera. For visualization purposes, the luminescent image (exposure time 1 minute) was overlaid on a photographic image. The signal intensity is represented by radiance and encoded by pseudo colors on the luminescent image.

### Flow cytometry

The iPSC-derived cells were analyzed using flow cytometer as described previously [12]. Briefly, these cells were harvested, washed, and suspended in cold phosphate-buffered saline containing 1% bovine serum album. The cells were then incubated with donkey serum to block nonsepecific binding, followed by serial incubations with Cy5-conjugated antibody for 1 hour at 4°C. The corresponding isotype antibodies were used as control. After incubation, fluorescence signals from Cy5 staining cells excited at 647 nm were collected at emission of >665 nm. The distributions of CD34, CD56, SM/C-2.6, M-cadherin or integrin-α7 expressing cells were analyzed using flowcytometer (FACS Canto, Becton Dickinson, CA).

### Histological analysis

Skeletal muscle samples were fixed in 4% paraformaldehyde, embedded in paraffin, and stained by H&E and picrosirius red for analysis of central nuclei and fibrosis. The central nuclei were quantified by measuring in the nucleated transverse section of the skeletal muscle cells. To determine collagen deposition, sections stained with picrosirius red were scanned and analyzed with a digital image analyzer. Collagen fractions were calculated as the ratio of the collagen area and the total muscle area in the corresponding section.

### Immunostaining

Immunostaining was performed on limb skeletal muscle tissue at 4 weeks post cell engraftment. Skeletal muscle tissue was fixed in 4% paraformaldehyde and prepared in sections at 5-μm thickness. Dystrophin mouse monoclonal antibody (Sigma-Aldrich), acetylcholine receptor rabbit polyclonal antibody (Sigma-Aldrich), and Pax-7 antibody were applied on the sections to assess the corresponding protein signals, and 4’, 6-diamino-2-phenyindole (DAPI, Sigma) was used to identify cell nuclei. Fluorescence labeled secondary antibodies (Jackson Immuno Research laboratories) were used, following these primary antibodies. Fluorescent imaging was performed with the Olympus BX41 microscope (Olympus America Inc., Melville, NY, U.S.A) equipped with epilflourescence attachment, and images were recorded using a digital camera with MagnaFire2.1 software.

### Statistical analysis

Data were expressed as mean ± SEM. Comparisons between two groups were evaluated by Student’s t-test (SPSS 13.0. IBM Co., Armonk, NY). Differences were considered to be significant at values of p<0.05.

## Results

### Reprogramming of mouse fibroblasts to generate iPSCs

**Figure 1:**
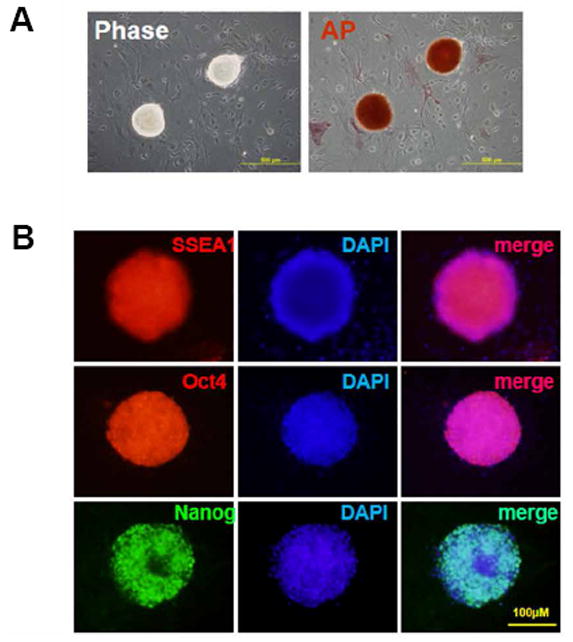
Characterization of mouse fibroblast-derived iPSC. (A) iPSCs colonies exhibited typical stem cell morphology (left) and showed high alkaline phosphatase (AP) activity (right). (B) iPSCs express SSEA1, Oct4 and Nanog as shown by immune-fluorescent staining.

Fibroblasts were isolated and purified from mouse ear tissue, and the lentivirus-containing Oct-4, Sox2, Klf4, c-Myc-encoding genes were then infected into fibroblasts to initiate fibroblast reprogramming. As shown in Figure 1A, the iPSCs colonies were observed between 25 and 30 days post transfection, and these induced-stem cells were further confirmed by alkaline phosphatase staining. Importantly, the pronounced expressions of Oct-4, Stage-Specific Embryonic Antigen-1 (SSEA1) and Nanog were detected in these colonies (Figure 1B), suggesting that pluripotency was successfully acquired by these reprogrammed cells.

### Phenotypes of iPSC-derived MPCs

**Figure 2:**
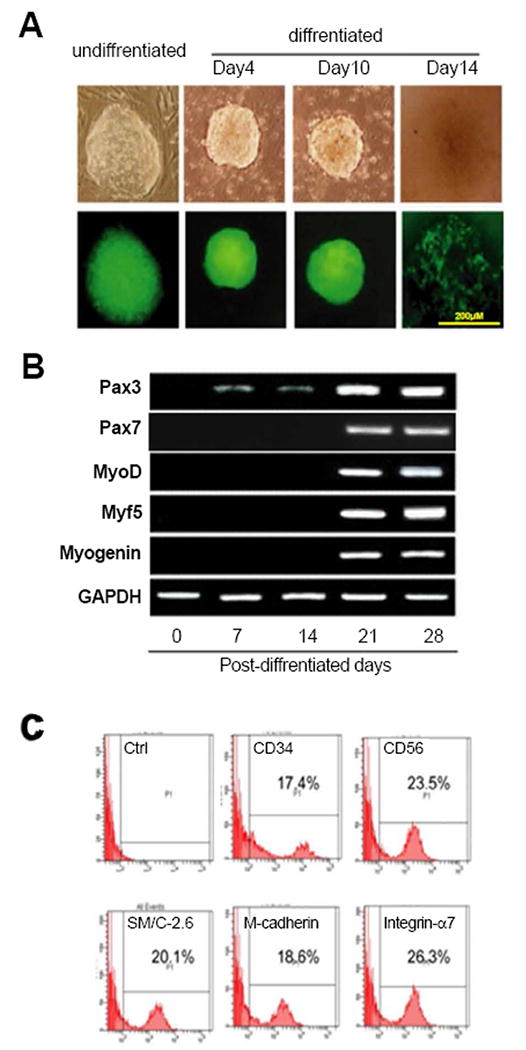
Generation of dystrophin overexpressing iPSC-derived myocyte progenitor cell (MPC) (A) Embryoid body of iPSC in undifferentiated and differentiated conditions. (B) Myogenic genes expression was assessed by PCR. Representative electrophoresis illustrates the expression levels of Pax-3, Pax-7, MyoD, Myf5, Myogenin, and GAPDH. (C) Flow cytometry was performed to determine the distribution of CD34, CD56, SM/C-2.6, M-cadherin, and integrin- α7.

Myogenic progenitor cells which carry dystrophin gene and express associated proteins restored contractility. Thus, we genetically modified the iPSCs with vector encoding GFP under the control of α-skeletal actin promoter. As expected, the green fluorescence was detected 100% in Embryoid Bodies (EB), suggesting that the α-skeletal actin promoter was successfully recombined into the iPSCs genome (Figure 2A). During the course of differentiation, the size of EB, as well as green fluorescence were decreased in a time dependent manner. At day 14 post-differentiation, the EBs in suspension disappeared and the iPSCs began to grow and were attached to the surface (Figure 2A). Accordingly, at 0~14 days during differentiation period, there was no expression of skeletal muscle-related genes, including Pax-7, Myo-D, Myf5 and myogenin, except weak expression of Pax-3 gene was observed (Figure 2B). The PCR gel electrophoresis analysis indicated that these muscle-related genes were expressed strongly after 21 days in differentiation medium. Furthermore, the profiles of myogenic stem/progenitor cell related surface markers were analyzed by flowcytometry, and comprised of CD34, CD56, SMC-2.6, M-cadherin and integrin-α7 with percentages of 17.4%, 23.5%, 20.1%, 18.6% and 26.3% respectively (Figure 2C). Overall, these results suggested that iPSCs can differentiate into myocyte progenitor cells post the transduction of α-skeletal actin promoter.

### Purification of MPC from iPSC-derived cells

**Figure 3:**
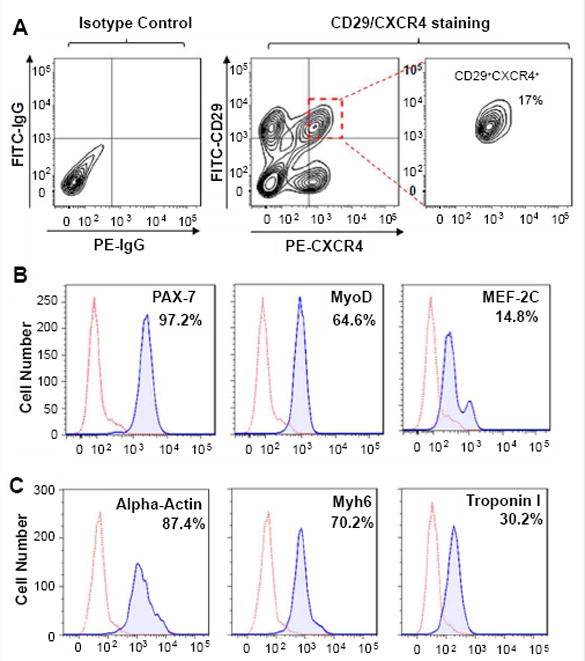
Myogenic progenitor cells were purified from iPSC-derived cells. (A) CD29^+^CXCR4^+^ cell population was identified as MPCs, which was sorted from iPSC-derived cells by flow cytometer. (B) Expressions of Pax-7, MyoD and MEF-2C in sorted-MPCs were analyzed by flow cytometer. (C) Flowcytometry analysis indicated the expressions of alpha-actin, Myh6 and troponin I in MPCs-differentiated cells.

To further investigate the therapeutic effects of iPSC-derived MPCs on muscular dystrophy, MPCs was fractioned and purified from iPSC-derived cells, using Fluorescent-Activated Cell-Sorting (FACS). The previous study revealed that MPCs are characterized by the pronounced co-expression of CD29 and CXCR4. As is shown in Figure 3A, the MPCs population has taken up 17% of iPSCs-differentiated cells, associating with intensified signals of Pax-7 (97.2%) and MyoD (64.6%) (Figure 3B). As the critical transcription factor in regulating skeletal muscle development during advanced stage, the signal of MEF-2C was detected in 14.8% iPSC-derived cells (Figure 3B). Followed by another 20 days’ differentiation, these MPCs were successfully differentiated into skeletal muscle cells, as evidenced by the expressions of alpha-actin (87.4%), myosin heavy chain 6 (70.2%), troponin I (30.2%) (Figure 3C).

### The viability of iPSC-MPCs *in vivo*

**Figure 4:**
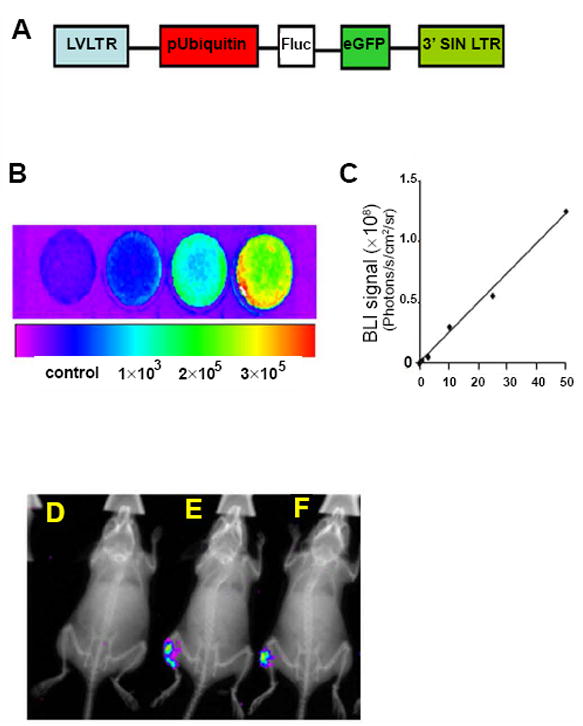
*In vivo* tracking of iPSC-derived MPC with BLI and x-ray overlay post transplantation. (A) A lentiviral vector carrying a double fusion (DF) reporter gene with the ubiquity promoter driving both firefly luciferase (Fluc) and EGFP was constructed and transduced into iPSC-derived MPC. (B) Bioluminescent imaging (BLI) in iPSC-MPCs plates. (C) Linear correlation of cell numbers and BLI signals (photons/s/cm2 per steridian) (D) An example of in vivo BLI is shown for mice implanted with iPSC-MPCs without reporter gene as background control (E,F) Mice implanted with iPSC-MPCs that transduced with firefly luciferase reporter gene.

The tissue microenvironment of dystrophic skeletal muscle is featured by the infiltration of immune cells, induction of inflammatory cytokines [15], respiratory insufficiency, and chronic hypoxia [16], and these detrimental biological effects can jeopardize the fate of the transplanted progenitor cells. To investigate the survival of the engrafted iPSC-MPCs, ubiquitin promoter-driven firefly luciferase (Fluc) expression vector (Figure 4A) was transduced into iPSC-MPCs, and the cell viability and survival were then evaluated by assessing luminescence intensity in cells after exposure to luciferase substrate. As shown in Figure 4B, the most intensive firefly luminescence signal was observed in culture dish with highest cell number (3×10^5^ cells/dish), while there was no signal detected in the control culture dish (no seeded cells), revealing a positive correlation between the amount of viable cells and luminescence signal intensity. Indeed, a standard curve was prepared (Figure 4C), by which the number of transplanted viable iPSC-MPCs can be quantified using luminescence signal intensity. Accordingly, firefly luminescence-expressing iPSC-MPCs were transplanted into cardiotoxin-injured skeletal muscle. As indicated by X-ray image-combined luminescence, signal (Figures 4D-4F) obtained after administration with luciferase, there is no luminescence detected in mouse muscle tissue that implanted with iPSC-MPCs without luminescence reporter gene (Figure 4D). Interestingly, the pronounced luminescence signal was observed in left limb in which the firefly luminescence-expressing iPSC-MPCs were engrafted (Figures 4E and 4F). Importantly, this signal remained at 30-days post progenitor cells’ transplantation, suggesting that the engrafted progenitor cells survived in the dystrophic muscle.

### IPSC-derived myogenic progenitor cells ameliorate muscular dystrophy in Mdx mouse

**Figure 5:**
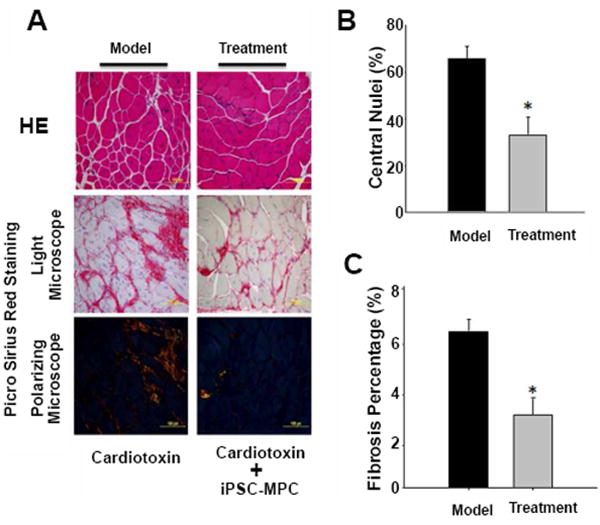
Central nuclei and fibrosis in mdx mice. (A) The histological sections of MDX mice anterior muscle stained with hematoxylin and eosin (H&E), picrosirus red. Nuclei centralization and fibrosis were investigated using light and polarized microscope. (B, C) Quantitative analysis indicated that treatment with dystrophin overexpressing iPSC-derived MPC improved pathologies associated with cardiotoxin injury, as evidence of reduced percentage of central nuclei muscle fibers (B), but significantly decreased tissue-fibrosis in anterior muscle. (n=5 each group, *P<0.05 *vs.* Model group).

An increase in the number of central nuclei is one of prominent features in the development of muscular dystrophy, which is caused by the loss of dystrophin. Indeed, quantitative analysis of the H&E stained muscle tissue indicated that the central nuclei were not observed in tibialis muscle in WT mouse under normal conditions (data not shown). In the tibialis muscle of mdx mouse, the percentage of central nuclei was increased to 64% post cardiotoxin administration. Interestingly, treatment with iPSCs-derived myogenic progenitors significantly decreased the central nuclei percentage to 35% (Figures 5A and 5B).

Fibrosis is another feature in the development of DMD due to muscle cell necrosis and inflammatory processes. As is shown in Figure 5A, amount of fibrous connective tissue increased in cardiotoxin injured tibialis muscular tissue, as evidenced by the picrosirius red staining (Figure 5A). The quantitative analysis showed that the fibrotic tissue was made up 6.3% of the whole muscular mass (Figure 5C) and fibrosis was significantly decreased by iPSC-MPCs treatment. These data suggest that iPSC-MPCs treatment can alleviate the pathological features of muscular dystrophy.

### IPSC-MPCs treatment restores dystrophin distribution and acetylcholine receptor expression in skeletal muscle

**Figure 6:**
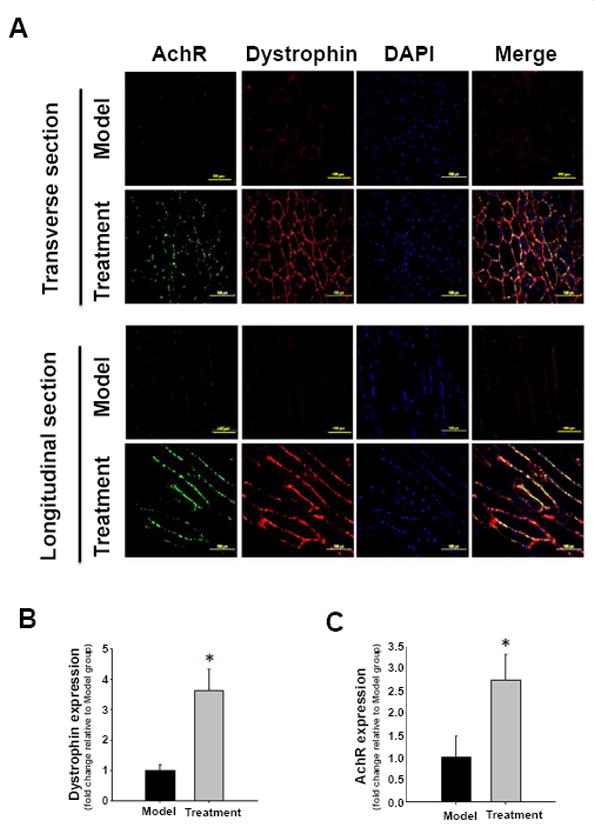
Expressions of dystrophin and acetylcholine receptor in the tibialis anterior muscle of MDX mice. (A) Acetylcholine receptors (AchR) were stained with AlexaFluo488-conjugated bungarotoxin (green) and the dystrophin was stained by TRITC-conjugated antibody (red). (B,C) quantitative analysis indicated that treatment with dystrophin overexpressing iPSC-derived MPC enhanced the expression levels of dystrophin (B) and AchR (C) in mdx mouse skeletal muscle post cardiotoxin injury. (n=5 each group, *P<0.05 *vs.* Model group).

Dystrophin is a critical protein that connects the muscle fiber cytoskeleton to the surrounding extracellular matrix through the cell membrane. From both transverse and longitudinal sections, fluorescent immunostaining revealed that the distribution of dystrophin in pericellular membrane was reduced in mdx muscle post cardiotoxin injection, while administration of iPSC-MPCs restored the distribution of this matrix structural protein (Figure 6A). Dystrophin expression was increased 3.5-fold in the iPSC-MPCs-treated muscular tissue when compared to non-treatment muscle (Figure 6B). Nicotinic Acetylcholine Receptor (nACHR) is a neuron receptor that induces skeletal muscle contraction upon binding and stimulation of neurotransmitter acetylcholine. The co-localization of nACHR and dystrophin has been reported by previous studies. As is shown in Figure 6A, the disposition of nachr, as well as its co-localization with dystrophin, was remarkably decreased in cardiotoxin-injured muscle tissue. This degraded protein complex was significantly restored with the treatment of iPSC-MPCs, as shown by the increased expression of nACHR (Figure 6C) and its colocalization with dystrophin (Figure 6A). These results suggested that administration of iPSC-MPCs promoted the restoration of dystrophin and nachr in dystrophic skeletal muscle.

### Pax-7 expression in dystrophic muscle post iPSC-MPCs treatment

**Figure 7:**
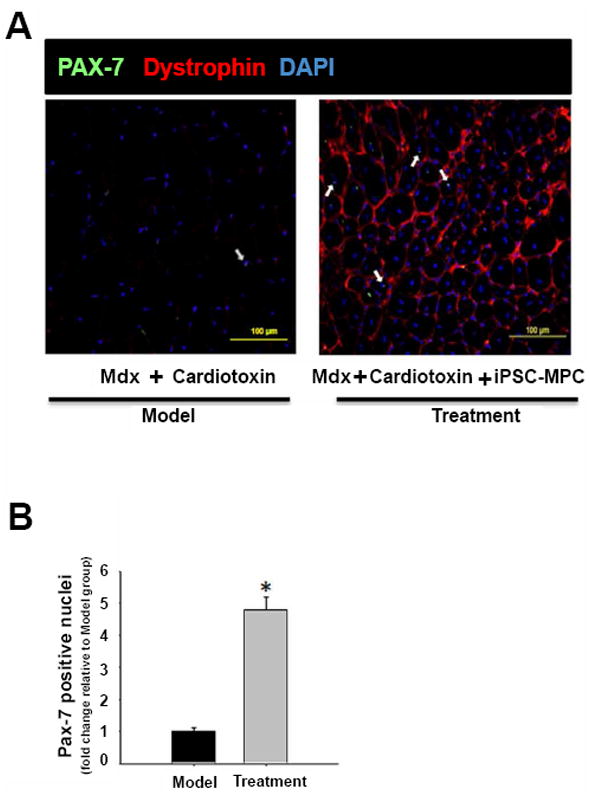
Treatment with dystrophin overexpressing iPSC-derived MPC promotes the expression of Pax-7 in the skeletal muscle. (A) Pax-7 and dystrophin in tibialis anterior muscle were stained with specific antibody and shown in green (FITC) and red (TRITC) fluorescence, respectively. The co-localization of green and DAPI (blue fluorescence) was identified as the pax-7 positive nuclear, which was indicated by white arrows. (B) Quantitative result of Pax-7 positive nuclei. (n=5 each group, *P<0.05 *vs.* Model group).

Muscle progenitor cells have been reported as a crucial component of satellite cell pool responsible for bestowing robust regeneration capacity of adult skeletal muscle [17]. These satellite cells uniformly express the transcription factor Pax7, which is the major factor in biogenesis of myogenic precursors. It maintains the viability of progenitor cells by activating target genes such as Myf5 and MyoD [18]. The green immunofluorescence staining of Pax7 was mainly confined to cell nuclei (Figure 7A). When mdx muscle was injured by cardiotoxin, Pax7 expression was reduced concomitant with loss of dystrophin (Figure 7A). Interestingly, the myogenesis was highly pronounced in cardiotoxin injured mdx muscle tissue after treatment with iPSC-MPCs, as supported by a 4.5-fold increase in Pax7 positive muscle nuclei (Figure 7B) suggesting that Pax7 expression is important in myogenesis of dystrophic muscle.

## Discussion

Muscular dystrophy is a recessive muscular disorder clinically featured by progressive muscle wasting and weakness. Duchenne muscular dystrophy is an X-chromosome-linked genetic disease, which is characterized by the mutation and loss of dystrophin expression. Indeed, dystrophin maintains the integrity of muscle fiber by connecting cytoskeleton to the surrounding extracellular matrix [19]. Many therapeutic strategies, including embryonic stem cell and adult stem cells transplantation, have been attempted to overcome the muscle degeneration and feebleness. The progress to date is limited. In this study, we reported that engraftment of iPSCs-derived MPCs restored dystrophin distribution and attenuated fibrosis and fixed central nucleus anomaly of dystrophic fiber.

The harsh microenvironment develops in the dystrophic muscle due to hypoxia, inflammation, and this has been considered to have significant impact on the therapeutic effects of transplanted stem cells. Indeed, dystrophin deficiency is associated with a disparate, pathological hypoxic stress response, contributed by the ongoing muscle necrosis in respiratory muscles [16]. Notably, the vicious continuous cycle of myofibers necrosis and repair continues in DMD muscle. The ongoing myofiber’s degeneration and necrosis can produce Th1 inflammatory stimuli to recruit neutrophils and monocytes/macrophages. Although these inflammatory cells can engulf and clear cell debris, the detrimental cytokines, such as tumor necrosis factor-alpha, are released to induce further muscle membrane damage threatening the survival of transplanted progenitor cells. Following Th1 stimuli, Th2 immune response will follow to promote muscle healing allowing participation of M2 macrophage expressing arginase-1, CD163, and CD206. Such a population of immune cells can produce Interleukin-10 (IL-10), Interleukin-17 (IL-17), Transforming Growth Factor β1 (TGF β1) to promote the resolution of inflammation and muscle healing [20]. However, the dysregulated wound healing can result in tissue fibrosis [21], whereby both the extracellular matrix and scarring impair proper skeletal muscle function and constitutes a barrier to progenitor cells’ integration into the injured muscle.

In present study, despite the inhospitable tissue microenvironment in DMD muscle, we provided the direct *in vivo* evidence, by bioluminescent imaging, that iPSC-derived MPCs can survive and exert beneficial effects to promote the regeneration of DMD muscle. Indeed, *in vitro* evidence of myogenesis was shown in iPSCs exposed to myogenic differentiation conditions by showing expression of Pax3 and Pax7. The Pax gene family is composed of transcription factors, which play crucial role in regulating myogenesis and maintaining tissue homeostasis. Notably, there is a synergistic functional crosstalk between Pax3 and Pax7, both involved in the maintenance of skeletal muscle progenitors. Pax3 expression is crucial for delamination and migration of muscle progenitors from somites to the limbs, while Pax7 plays a dual role in the regulation of myogenesis by activating myogenic program and simultaneously preventing terminal differentiation. Specifically, upon receiving stimuli from injured muscle, Pax7 expression will quickly promote the entry of quiescent satellite cell into myogenic program by activating MyoD. Subsequently, MyoD will induce the expression of myogenin to direct the terminal differentiation in myogenic progenitor cells through up-regulation of the muscle specific genes. At the same time, the accumulated Pax7 can blockade MyoD signaling pathway to activate myogenin, and this negative feedback mechanism can keep Pax7-positive progenitors from draining out. Actually, it is exciting to observe the Pax7 expression in DMD muscle post iPSC-MPCs transplantation which is strong indication for initiation of myogenesis. The present study demonstrated that the enhanced dystrophin expression was associated with the Pax7’s up-regulation. As a hydrophobic adaptor protein, dystrophin provides a connection between intracellular cytoskeleton and the surrounding extracellular matrix, which is crucial component in maintaining muscle movement. Interestingly, the amount of acetylcholine receptors increased in DMD muscle tissue post transplantation of iPSC-MPCs, suggesting that neuromuscular regulation has been improved in dystrophic tissue.

In summary, the committed myogenic differentiation was successfully induced by iPSCs-derived MPC. Importantly, iPSC-derived MPC significantly decreased the frequency of central nuclei and mitigated the tissue fibrosis in DMD muscle which was accompanied by enhanced Pax7 expression, redistribution of dystrophin, and reconstitution of acetylcholine receptors thus suggesting a strong therapeutic potential of iPSCs derived muscle progenitors in the treatment of muscular dystrophy.

## Supplementary Material

Supplementary file
