# Supplementary material for: Induced Pluripotent Stem Cells derived Muscle Progenitors Effectively Mitigate Muscular Dystrophy through Restoring the Dystrophin Distribution
Source: J Stem Cell Res Ther. Author manuscript; Available in PMC 2017 Oct 2. (PMC5624556; doi:10.4172/2157-7633.1000361)
Supplement: Supplementary file [file NIHMS829464-supplement-Supplementary_file.doc]

**Table 1: Primers Sequences**

| Name | Forward | Reverse |
| --- | --- | --- |
| Pax-3 | 5’-AAACCCAAGCAGGTGACAAC-3’ | 5’-CCTCAGGATGCGGCTGATAG-3’ |
| Pax-7 | 5’-GAAAGCCAAACACAGCATCG-3’ | 5’-CTGATTCCACATCTGAGCCC-3’ |
| MyoD | 5’-TGGCATGATGGATTACAGCG-3’ | 5’-GAGATGCGCTCCACTATGCT-3’ |
| Myf5 | 5’-TGACGGCATGCCTGAATGTAAC-3’ | 5’-CTCGGATGGCTCTGTAGACG-3’ |
| Myogenin | 5’-CGGCTGCCTAAAGTGGAGAT-3’ | 5’-GCGAGCAAATGATCTCCTGGG-3’ |
| GAPDH | 5’-CCCTTAAGAGGGATGCTGCC-3’ | 5’-TACGGCCAAATCCGTTCACA-3’ |
